# Knowledge, Attitude, and Practice Regarding COVID-19 Among Healthcare Workers in Primary Healthcare Centers in Dubai: A Cross-Sectional Survey, 2020

**DOI:** 10.3389/fpubh.2021.617679

**Published:** 2021-07-28

**Authors:** Abdulaziz Hussain Albahri, Shatha Ahmed Alnaqbi, Shahad Ahmed Alnaqbi, Asma Obaid Alshaali, Shaikha Mohammad Shahdoor

**Affiliations:** Primary Healthcare Services Sector, Dubai Health Authority, Dubai, United Arab Emirates

**Keywords:** COVID-19, SARS-CoV-2, KAP study, primary healthcare, fever clinic, family physician, Dubai, UAE

## Abstract

**Introduction:** The coronavirus disease 2019 (COVID-19) pandemic continues to challenge healthcare services worldwide. Healthcare workers (HCWs) are key to the continued effort to overcome the pandemic. This study aims to evaluate the knowledge, attitude, and practices of HCWs toward COVID-19 in primary health centers in Dubai.

**Methods:** This cross-sectional study was conducted at four primary health centers in Dubai, including two fever clinics, from July 5th to July 11th, 2020. A self-administered online questionnaire was distributed to nurses and physicians working in these centers, which evaluated their knowledge, attitude, and practices regarding COVID-19 and their associations with the participants' demographic factors. A total score of 80% and above constituted a level of sufficiency in each section. Additionally, Mann-Whitney U test and multivariable logistic regression were used to analyze the variables.

**Results:** A total of 176 HCWs completed the questionnaire, with a 91.2% (176/193) response rate. They were predominantly female (158/176, 90.0%), nurses (128/176, 72.7%), and non-Emiratis (150/176, 85.2%). While official health organizations were the primary source of information for 91.5% (161/176) of participants, only 38.1% (67/176) reported using scientific journals as one of their sources. Overall, 57.4% (101/176) of participants had a sufficient overall level of knowledge. Moreover, knowledge regarding signs, symptoms, and at-risk groups was generally satisfactory. However, knowledge about the virus, testing, transmission, and the isolation of contacts with positive cases was identified correctly by less than two-thirds of the participants. Half of the participants (89/176, 50.6%) expressed their concern about personally acquiring the infection, 112/176 (63.6%) worried about their relatives acquiring it, and 72/176 (40.9%) expressed some hesitancy to take the COVID-19 vaccine once available. Overall, only 58/176 (33.0%) HCWs had a sufficient overall positive attitude score. Nurses, compared to physicians, and non-Emiratis compared to Emiratis' HCWs, had statistically higher mean scores for attitude (*U* = 2,212, *p* < 0.01; and *U* = 1164.5, *p* < 0.01, respectively). The majority of participants (156/176, 88.6%) reported acceptable infection control practices.

**Conclusion:** Given the gaps identified in the knowledge and attitude, we recommend further training to improve the skills of primary HCWs, with encouragement to practice evidence-based medicine. Additionally, further exploration regarding vaccine hesitancy is warranted.

## Introduction

The world has faced a significant challenge since the outbreak of the novel coronavirus in China in December 2019. Healthcare systems worldwide struggled to cope with the many patients suffering from the novel coronavirus disease 2019 (COVID-19), with shortages in medical supplies and medical staff commonplace (1). COVID-19 is an infection caused by severe acute respiratory syndrome-coronavirus-2 (SARS-CoV-2), which is a beta coronavirus that was first detected in Wuhan, China. It rapidly spread globally, causing more than 34 million infections and over one million deaths by October 2020 (1, 2). The virus spreads primarily via droplets and close contact with infected individuals, with the most common symptoms including fever, dry cough, difficulty breathing, and myalgia (1, 3). However, some less common symptoms exist, such as gastrointestinal upsets and loss of smell or taste (1). The severity of these symptoms varies from person to person, but being elderly and having comorbidities are risk factors for more severe diseases (1, 3). As of October 2020, treatment is focused on the symptomatic management of patients, with hospitalization required for more severe cases. Additionally, some experimental medications are being trialed with various efficacies (1). Once more, as of October 2020, there is no available vaccine for COVID-19; however, multiple candidates are undergoing clinical trials, which might be available by the end of the year (4). It is recognized that healthcare workers (HCW) worldwide are at a higher risk of contracting the infection (5, 6). However, infection control measures, such as hand hygiene, social distancing, and proper donning and doffing of personal protective equipment (PPE), have proven to lessen the spread of the infection (5).

The United Arab Emirates (UAE), and Dubai in particular, saw its first case of COVID-19 infection on the 29th of January 2020. By March, the number of daily reported cases rose steeply, leading the local government to institute a 24-h lockdown in early April. By July 2020, there were over 50,000 reported cases and over 300 deaths (2, 7). Dubai Health Authority (DHA) had designated two of its 12 primary healthcare centers (PHCs) as fever clinics to direct the flow of suspected COVID-19 patients away from regular clinics.

The governmental healthcare system in Dubai before the pandemic was composed of 12 PHCs, where family physicians saw patients face-to-face in a mixed system of walk-ins and by appointment. However, during the pandemic, there was a restructuring of the PHCs. One of the centers was converted into a child health and antenatal care center. Another two functioned as 24-h walk-in centers, two fever clinics, and two clean clinics where no fever cases or suspected COVID-19 instances had been seen. The rest remained business as usual, redirecting any suspected COVID-19 cases to the fever clinics and minimizing face-to-face contact by increasing the utilization of the telemedicine service. The purpose of the fever clinics was to redirect any suspected COVID-19 case from any of the other PHCs to a specialist service, where patients would get a COVID-19 PCR swab and overall evaluation about the stability of the case and initiation of symptomatic treatment as indicated. Confirmed positive cases would then be redirected to isolation centers, field hospitals, or main central hospitals based on the severity of the case.

By the second half of June, Dubai and the UAE, in general, had passed its first peak of the outbreak, and the lockdown had primarily been eased, with businesses being encouraged to resume work under new normality (7). By that time, the possibility of a second wave of infection had been speculated by some international experts; therefore, evaluation of the healthcare status in the city was of utmost importance for the preparation to overcome any future related healthcare challenges (8, 9). HCWs play a crucial role in controlling and managing any potential new outbreaks or resurgence of the infection. Therefore, the current study aimed to evaluate the knowledge, attitude, and practices (KAP) concerning COVID-19 among HCWs working in PHCs in Dubai to overcome any existing or future challenges. Additionally, lessons learned from this study might shed some light and provide guidance to healthcare systems in the region and elsewhere to overcome similar difficulties.

## Methods

### Study Setting and Population

This cross-sectional study was conducted between July 5th and July 11th, 2020. The questionnaire was distributed to all family physicians and nurses working in two fever clinics and two regular PHCs in DHA. The focus was on HCWs in direct contact with patient care; thus, other staff, such as pharmacists and radiographers, were excluded. Additionally, nurses and family physicians working in other PHCs were omitted from the study. The selection of the health centers was based on the staff working in these centers being more stable throughout the first outbreak and reflected the practice in a more consistent environment compared to other centers that had variable staff who were shuffled between centers and responsibilities during that period.

The sample size was calculated using the CDC Epi Info v7.2.4 software (accessible at: https://www.cdc.gov/epiinfo/) using a single population proportion formula with the assumptions of 95% confidence level, 3% margin of error, 50% proportion of insufficient knowledge, and a total population of 193 HCWs practicing in the four health centers. Accordingly, the minimum sample size required for the study was 164.

### Study Questionnaire and Data Collection

An online-based, self-administered questionnaire was constructed based on a literature review of previously published relevant questionnaires and in keeping with the World Health Organization (WHO) recommendations (1, 3, 10–16).

The questionnaire was comprised of four sections: demographic data, knowledge, attitude, and practice sections. The demographic section asked the participants for their gender, age, nationality, profession and professional experience, workplace, and sources of COVID-19 information. The knowledge section had 15 questions that assessed the participants' knowledge on COVID-19 etiology, signs and symptoms, treatment and management, prevention, transmission, and risk factors. Each question had the answer options of true, false, or I don't know. Only the correct answer was given a score of 1; all other answers scored 0. Therefore, the total score for the section ranged from 0 to 15. The attitude section had seven questions, evaluating the participants' level of fear from COVID-19, their willingness to take preventive measures, help on the frontlines, be isolated if infected, and their confidence in beating the pandemic. Only the single answer that indicated a positive attitude was given a score of 1; other answers scored 0, with a total score ranging from 0 to 7 for the entire section. Finally, the practice section consisted of six questions evaluating the participants' infection control measures during the outbreak. Measures that were always practiced were given scores of 1; otherwise, they scored 0. Accordingly, the maximum score for this section was 6.

To have a sufficient score in each section, Bloom's cutoff point of 80% was selected (17). Therefore, a score of 12 and above was considered having sufficient knowledge, 5.6 and above was a positive attitude, and 4.8 and above was good infection control practice. While some studies set the sufficient score at 70%, we used a more stringent criterion because of the seriousness of the disease and the importance of having a higher degree of knowledge, attitude, and practice to protect the staff, patients, and the community.

The questionnaire was piloted on 40 healthcare professionals and further modified to suit the local setting. Results from the pilot study were excluded from the final analysis. Face and content validities were examined by specialists in the field. A sample of the questionnaire is provided in the Supplementary Material.

The link to the online questionnaire was forwarded to the managers in charge of each health center, and they were asked to distribute it to all of their relevant staff via email or online messaging services. The link contained an explanatory page of the study and an informed consent form; once accepted, the participant was taken to the questions section to be completed.

### Statistical Analysis

Data were analyzed using GraphPad Prism v8.4.3, and Mann-Whitney *U*-test was used to evaluate the association between the demographic variables and the mean scores of the KAP. Pearson's correlation was used to assess the correlation between the knowledge score and the attitude and practice scores. Fisher's exact test was used to evaluate differences in knowledge between fever and regular clinics. Multivariable logistic regression was used to assess the association between the demographic variables and each character in the attitude and practice sections. Alpha <0.05 was deemed statistically significant. Raw data for the results are accessible at: https://figshare.com/s/27f23a6fd98149070a67.

### Ethical Approval

The DHA Scientific Research Ethics Committee approved this study (reference number: DSREC-06/2020_34). Participation in the survey was voluntary, and online informed consent was obtained before participation in the study.

## Results

There were 176 participants, with a survey response rate of 91.2% (176/193). They were predominantly female and non-Emirati HCWs [158/176 (90.0%) and 150/176 (85.2%), respectively]. More than two-thirds were nurses (128/176, 72.7%), 105/176 (60%) were age 40 and above, and 74/176 (42%) had more than 15 years of experience. There was an almost equal distribution regarding the workplace, with 49.4% (87/176) of participants working in fever clinics and 50.6% (89/176) in regular clinics. The data are summarized in Table 1.

**Table 1:** Baseline demographic characteristics of participants (*n* = 176).

**Characteristic**		**No. (%)**
**Age**		
	<40	71 (40.0)
	≥40	105 (60.0)
**Gender**		
	Female	158 (90.0)
	Male	18 (10.0)
**Nationality**		
	Emirati	26 (14.8)
	Other	150 (85.2)
**Profession**		
	Nurse	128 (72.7)
	Physician	48 (27.3)
**Years of experience**		
	≤ 15	102 (58.0)
	>15	74 (42.0)
**Workplace**		
	Fever clinic	87 (49.4)
	Regular clinic	89 (50.6)
**Primary source of information**		
	Official health organizations	161 (91.5)
	Social media	51 (29.0)
	News media	77 (43.8)
	Scientific journals	67 (38.1)
	Work colleagues	73 (41.5)
	Seminars and workshops	64 (36.4)
	Internet	69 (39.2)
	Other	11 (6.3)

The majority of participants reported that official health organizations' resources were their primary source of information regarding COVID-19 (161/176, 91.5%). This was followed by news media and work colleagues, with 43.8% (77/176) and 41.5% (73/176), respectively. Only 38.1% (67/176) of respondents stated scientific journals and research papers as their main sources of information. Seminars and/or workshops were among the primary sources for 36.4% (64/176) of participants. On the other hand, only 51/176 respondents (29.0%) stated social media as their primary source of information. The internet was a primary source for 69/176 (39.2%) respondents, as summarized in Table 1.

### Knowledge

The mean score for the knowledge section was 11.78 (*SD* = 1.34), with only 101/176 (57.4%) of participants having sufficient knowledge at a cutoff point of 80% of the total score. The data for each question is summarized in Table 2. The least correctly answered question was regarding the diagnosis of the illness using PCR. The test detects the RNA of the virus and not its proteins, with only four participants selected the correct answer. The other least scored questions were (with the correct answer for each being false): the period of observation for a contact with a confirmed case being 28 days, the name of the virus being SARS-CoV-1 instead of SARS-CoV-2, and that early antibiotic use shortened the duration of the illness, with 36.9% (65/176), 44.9% (79/176), and 63.1% (111/176), answering the questions correctly, respectively. Additionally, only 80.7% (142/176) answered correctly that wearing a mask by the general public could prevent them from acquiring the infection. All other knowledge questions were answered correctly by at least 89.2% (157/176) of participants, as summarized in Table 2.

**Table 2:** Knowledge, attitude, and practice scores of healthcare workers regarding COVID-19 (*n* = 176).

**Question**	**Correct answer**	**No. (%)**	**Mean score (*SD*)**
**Knowledge**			
K1. There is currently no effective cure for COVID-19, but early symptomatic and supportive treatment can help most patients recover from the infection.	True	171 (97.2)	
K2. Not all persons with COVID-19 will develop severe cases. Those who are elderly, have chronic illnesses, and are obese are more likely to be severe cases.	True	167 (94.9)	
K3. Persons with COVID-19 cannot transmit the virus to others when a fever is not present.	False	157 (89.2)	
K4. The COVID-19 virus spreads via respiratory droplets of infected individuals.	True	170 (96.6)	
K5. Wearing general medical masks by the public can prevent one from acquiring infection by the COVID-19 virus.	True	142 (80.7)	
K6. It is not necessary for children and young adults to take measures to prevent the infection by the COVID-19 virus.	False	166 (94.3)	
K7. To prevent the infection by COVID-19, individuals should avoid going to crowded places such as bus parks and avoid taking public transportation.	True	166 (94.3)	
K8. Isolation and treatment of people who are infected with the COVID-19 virus are effective ways to reduce the spread of the virus.	True	172 (97.7)	
K9. People who have contact with someone infected with the COVID-19 virus should be immediately isolated in a proper place. In general, the observation period is 28 days.	False	65 (36.9)	
K10. Diarrhea is a possible symptom of COVID-19.	True	174 (98.9)	
K11. Currently COVID-19 vaccine is available in the market.	False	162 (92.0)	
K12. Healthcare workers are at a higher risk of infection.	True	168 (95.5)	
K13. Early antibiotic use shortens the duration of COVID-19 illness.	False	111 (63.1)	
K14. SARS-CoV-1 is the causative agent of COVID-19 infection.	False	79 (44.9)	
K15. Detection of the viral protein via PCR analysis of the patient's sample is the main way of diagnosing COVID-19.	False	4 (2.3)	
**Knowledge toward COVID-19** ^**a**^			11.78 (1.34)
*Sufficient*		101 (57.4)	
*Insufficient*		75 (42.6)	
Attitude	Positive attitude answer		
A1. You are extremely worried that you might catch the COVID-19 infection.	Disagree	87 (49.4)	
A2. You are extremely worried that one of your family members might get infected.	Disagree	64 (36.4)	
A3. If getting COVID-19, you will accept isolation in health facilities.	Agree	144 (81.8)	
A4. Prevalence of COVID-19 can be reduced by the active participation of healthcare workers in infection control programs.	Agree	166 (94.3)	
A5. If a COVID-19 vaccine was available, I would have it.	Agree	104 (59.1)	
A6. COVID-19 pandemic will be successfully controlled.	Agree	115 (65.3)	
A7. If the country needs you, you will be willing to help in the frontline rescue.	Agree	165 (93.8)	
Attitude toward COVID-19^b^			4.80 (1.43)
*Sufficient*		58 (33.0)	
*Insufficient*		118 (67.0)	
Practice	Good practice answer		
P1. During the outbreak, did you participate in training programs to increase/refresh your practice on infection control and COVID-19?	Always	126 (71.6)	
P2. During the outbreak, did you use sodium hypochlorite or 70% alcohol as surface disinfectant?	Always	166 (94.3)	
P3. During the outbreak, did you wash your hands before and after contact with your patients?	Always	175 (99.4)	
P4. During the outbreak, did you maintain social distance at work place?	Always	170 (96.6)	
P5. During the outbreak, did you follow the steps in doffing your PPE as per protocol?	Always	162 (92.0)	
P6. During the outbreak, did you wear surgical mask for routine patient contact?	Always	171 (97.2)	
Practice related to COVID-19^c^			5.51 (0.76)
*Sufficient*		156 (88.6)	
*Insufficient*		20 (11.4)	

a *Sufficient knowledge per participant is considered at a total score of 80% and higher (12 points and above)*.

b *Sufficient attitude per participant is considered at a total score of 80% and higher (5.6 points and above)*.

c *Sufficient practice per participant is considered at a total score of 80% and higher (4.8 points and above)*.

When comparing individual items in the knowledge section between fever and regular clinics, there was no statistically significant difference between the two groups except for the question that general mask use by the public could protect one from acquiring the disease, with a higher proportion of participants in the fever clinic answering the question correctly [87.4% (76/87) vs. 74.2% (66/89), respectively, *p* < 0.05], as detailed in Table 3. The total knowledge score had a statistically significant association with nationality (*U* = 1,471, *p* < 0.05), with Emiratis having a higher mean score (mean = 12.27, *SD* = 1.15) vs. non-Emiratis (mean = 11.70, *SD* = 1.36). Additionally, physicians had a statistically significant higher score compared to nurses (*U* = 2338.5, *p* < 0.05), with a mean of 12.19 (*SD* = 1.48) vs. 11.63 (*SD* = 1.25) for nurses. There were no associations with other demographic variables (data summarized in Table 4). Moreover, there were no statistically significant correlations between the knowledge scores and the attitude or the practice scores (**Table 6**).

**Table 3:** Knowledge scores regarding COVID-19 amongst healthcare workers in fever compared to regular primary care clinics (*n* = 176).

	**Fever clinic (** ***n*** **= 87)**					**Regular clinic (** ***n*** **= 89)**				
**Question**	**Correct answer**			**Wrong answer**		**Correct answer**		**Wrong answer**		
		**No**.	**%**	**No**.	**%**	**No**.	**%**	**No**.	**%**	***P*-value**
**Knowledge**										
K1. There is currently no effective cure for COVID-19, but early symptomatic and supportive treatment can help most patients recover from the infection.	True	86	*98.9*	1	*1.1*	85	*95.5*	4	*4.5*	0.37
K2. Not all persons with COVID-19 will develop severe cases. Those who are elderly, have chronic illnesses, and are obese are more likely to be severe cases.	True	82	*94.3*	5	*5.7*	85	*95.5*	4	*4.5*	0.75
K3. Persons with COVID-19 cannot transmit the virus to others when a fever is not present.	False	79	*90.8*	8	*9.2*	78	*87.6*	11	*12.4*	0.63
K4. The COVID-19 virus spreads via respiratory droplets of infected individuals.	True	83	*95.4*	4	*4.6*	87	*97.8*	2	*2.2*	0.44
K5. Wearing general medical masks by the public can prevent one from acquiring infection by the COVID-19 virus.	True	76	*87.4*	11	*12.6*	66	*74.2*	23	*25.8*	0.035^*^
K6. It is not necessary for children and young adults to take measures to prevent the infection by the COVID-19 virus.	False	83	*95.4*	4	*4.6*	83	*93.3*	6	*6.7*	0.75
K7. To prevent the infection by COVID-19, individuals should avoid going to crowded places such as bus parks and avoid taking public transportation.	True	81	*93.1*	6	*6.9*	85	*95.5*	4	*4.5*	0.53
K8. Isolation and treatment of people who are infected with the COVID-19 virus are effective ways to reduce the spread of the virus.	True	86	*98.9*	1	*1.1*	86	*96.6*	3	*3.4*	0.62
K9. People who have contact with someone infected with the COVID-19 virus should be immediately isolated in a proper place. In general, the observation period is 28 days.	False	29	*33.3*	58	*66.7*	36	*40.4*	53	*59.6*	0.35
K10. Diarrhea is a possible symptom of COVID-19.	True	86	*98.9*	1	*1.1*	88	*98.9*	1	*1.1*	1.00
K11. Currently COVID-19 vaccine is available in the market.	False	77	*88.5*	10	*11.5*	85	*95.5*	4	*4.5*	0.10
K12. Healthcare workers are at a higher risk of infection.	True	82	*94.3*	5	*5.7*	86	*96.6*	3	*3.4*	0.49
K13. Early antibiotic use shortens the duration of COVID-19 illness.	False	53	*60.9*	34	*39.1*	58	*65.2*	31	*34.8*	0.64
K14. SARS-CoV-1 is the causative agent of COVID-19 infection.	False	36	*41.4*	51	*58.6*	43	*48.3*	46	*51.7*	0.37
K15. Detection of the viral protein via PCR analysis of the patient's sample is the main way of diagnosing COVID-19.	False	2	*2.3*	85	*97.7*	2	*2.2*	87	*97.8*	1.00
**Knowledge toward COVID-19**										
		***Sufficient***		***Insufficient***		***Sufficient***		***Insufficient***		
		No.	%	No.	%	No.	%	No.	%	
		47	54.0	40	46.0	54	60.7	35	39.3	0.45

*Fisher's exact test, with p < 0.05 considered as statistically significant*.

*Sufficient knowledge per participant is considered at a total score of 80% and higher (12 points and above)*.

* *p < 0.05*.

**Table 4:** Characteristics of participants and their associations with the knowledge, attitude, and practice scores regarding COVID-19 (*n* = 176).

**Characteristic**	**Knowledge score**				**Attitude score**				**Practice score**			
	**Mean (*SD*)**	***U*-value**	**Z score**	***P*-value**	**Mean (SD)**	***U*-value**	**Z score**	***P*-value**	**Mean (SD)**	***U*-value**	**Z score**	***P*-value**
**Age**												
≥40	11.80 (1.42)				4.71 (1.43)				5.54 (0.77)			
<40	11.76 (1.22)	3650.5	0.23	0.82	4.93 (1.44)	3,430	−0.90	0.37	5.47 (0.73)	3382.5	0.84	0.40
**Gender**												
Female	11.78 (1.36)				4.79 (1.43)				5.53 (0.75)			
Male	11.78 (1.22)	1386.5	0.17	0.87	4.89 (1.53)	1393.5	−0.14	0.89	5.39 (0.78)	1,269	0.74	0.46
**Nationality**												
Emirati	12.27 (1.15)				3.96 (1.46)				5.19 (0.85)			
Other	11.70 (1.36)	1,471	−1.99	0.046^*^	4.95 (1.38)	1164.5	3.27	0.001^**^	5.57 (0.73)	1,461	2.04	0.041^*^
**Profession**												
Physician	12.19 (1.48)				4.33 (1.39)				5.23 (0.88)			
Nurse	11.63 (1.25)	2338.5	−2.43	0.015^*^	4.98 (1.42)	2,212	2.86	0.004^**^	5.62 (0.68)	2,330	2.46	0.014^*^
**Years of experience**												
>15	11.80 (1.40)				4.73 (1.44)				5.53 (0.80)			
≤ 15	11.77 (1.30)	3690.5	−0.25	0.80	4.85 (1.44)	3,631	0.43	0.67	5.50 (0.73)	3,614	−0.48	0.63
**Workplace**												
Fever clinic	11.74 (1.33)				4.82 (1.41)				5.44 (0.86)			
Regular clinic	11.83 (1.35)	3,641	0.68	0.50	4.79 (1.47)	3863.5	−0.02	0.98	5.58 (0.64)	3655.5	0.64	0.52

*Man-Whitney U-test, with p < 0.05 indicating statistical significance*.

* *P < 0.05 and*

** *P < 0.01*.

### Attitude

Only 33% (58/176) of participants had a sufficient overall attitude score, with a mean score of 4.80 (*SD* = 1.43) for all the participants (Table 2). A significant number of participants expressed extreme worry about themselves or their family members getting infected, with only 36.4% (64/176) not worried about their relatives and 49.4% (87/176) not concerned about themselves. Additionally, only 59.1% (104/176) of respondents were willing to take the COVID-19 vaccine if available, and 65.3% (115/176) believed that the pandemic could be successfully controlled. Furthermore, 18.2% (62/176) expressed some hesitation or unwillingness in being isolated in a healthcare facility if they got infected. On the other hand, the majority of participants were willing to help on the frontlines and believed that the prevalence of the infection could be reduced by participation in infection control programs [93.8% (165/176) and 94.3% (166/176), respectively].

The attitude score had a statistically significant association with nationality (*U* = 1164.5, *p* < 0.01) and profession (*U* = 2,212, *p* < 0.01), with non-Emiratis and nurses having higher mean scores, as shown in Table 4. Multivariable logistic regression demonstrated that physicians and HCWs working in fever clinics were less willing to be isolated in a healthcare facility if they got infected compared to nurses and HCWs in regular clinics (aOR: 0.25, 95% CI: 0.08–0.80, *p* < 0.05; aOR: 0.27, 95% CI: 0.10–0.67, *p* < 0.01, respectively). Moreover, Emirati HCWs were less willing to be vaccinated against COVID-19 than HCWs from other nationalities (aOR: 0.23, 95% CI: 0.06–0.76, *p* < 0.05). Table 5 summarizes the logistic regression analysis of the attitude variables. Correlation analysis between the attitude scores and the practice scores showed a positive but weak correlation between the two [*r*_(174)_ = 0.2, 95% CI: 0.03–0.32, *p* < 0.05], suggesting that participants with higher positive attitude scores were more likely to also score higher in the practice part (Table 6). However, since the association was weak, and both sections were pure reporting of the participants rather than actual auditing in the field, this finding warrants further exploration in future studies.

**Table 5:** Multivariable logistic regression analysis of odds ratio for attitudes and practices of healthcare workers associated with their demographic characteristics (*n* = 176).

		**Variables, aOR (95% CI)**				
		**Age**	**Nationality**	**Profession**	**Years of experience**	**Workplace**
		**≥40 (vs. <40)**	**Emirati (vs. other)**	**Physician (vs. nurse)**	**>15 (vs. ≤15)**	**Fever clinic (vs. regular)**
**Attitudes**						
A1	Fear of catching the infection	0.65 (0.28–1.48)	0.72 (0.23–2.23)	0.83 (0.33–2.04)	1.39 (0.62–3.15)	1.40 (0.77–2.57)
A2	Fear about relatives being infected	0.63 (0.26–1.49)	0.41 (0.11–1.35)	1.15 (0.45–2.86)	1.35 (0.58–3.24)	0.81 (0.43–1.51)
A3	Willingness for isolation if infected	0.84 (0.28–2.60)	0.33 (0.09–1.20)	0.25^*^ (0.08–0.80)	1.66 (0.54–5.05)	0.27^**^ (0.10–0.67)
A4	Willingness to take part in infection control programs	1.32 (0.21–11.23)	0.22 (0.01–3.18)	4.74 (0.49–144.60)	0.55 (0.07–3.22)	2.32 (0.61–11.23)
A5	Willingness to be vaccinated	1.14 (0.47–2.83)	0.23^*^ (0.06–0.76)	0.61 (0.25–1.55)	0.52 (0.22–1.23)	1.10 (0.58–2.11)
A6	Positive about control of pandemic	1.08 (0.46–2.58)	1.06 (0.32–3.53)	1.18 (0.47–3.17)	0.82 (0.35–1.88)	0.93 (0.50–1.75)
A7	Willingness to help in frontline	0.30 (0.06–1.44)	1.12 (0.07–28.19)	1.81 (0.32–22.28)	2.36 (0.54–10.55)	1.32 (0.37–4.88)
**Practices**						
P1	Participation in infection control programs	1.99 (0.80–5.28)	1.01 (0.32–3.25)	0.37^*^ (0.14–0.99)	1.01 (0.38–2.57)	0.78 (0.39–1.53)
P2	Use of appropriate surface disinfectants	1.66 (0.26–14.10)	2.54 (0.25–57.30)	0.61 (0.13–4.08)	0.53 (0.07–3.02)	1.55 (0.42–6.31)
P3	Appropriate hand washing^#^					
P4	Social distancing at work	0.61 (0.05–10.33)	0.09 (0.001–6.33)	13.20 (0.41–1761.00)	1.48 (0.09–17.51)	0.43 (0.05–2.55)
P5	Proper doffing of PPE	0.74 (0.16–3.78)	0.94 (0.18–4.94)	0.19^*^ (0.04–0.83)	1.40 (0.28–6.29)	0.18^*^ (0.04–0.65)
P6	Appropriate use of surgical mask	0.32 (0.01–8.33)	0.02 (0.0002–0.49)	1.28 (0.03–78.32)	0.90 (0.04–9.80)	0.33 (0.03–2.46)

*aOR, adjusted odds ratio; CI, confidence interval*.

* *P < 0.05 and*

** *P < 0.01*.

# *Logistic regression is not applicable as all participants save for one selected the same positive answer*.

**Table 6:** Correlation between scores of knowledge, attitude, and practice (*n* = 176).

**Variable**	**Pearson's correlation coefficient**	***P*-value (95% CI)**
Knowledge-Attitude	0.088	0.25 (−0.06 to 0.23)
Knowledge-Practice	0.019	0.80 (−0.13 to 0.17)
Attitude-Practice	0.179^*^	0.018 (0.03 to 0.32)

* *Significant correlation at p < 0.05 (two-tailed)*.

### Practice

The majority of participants (156/176, 88.6%) reported an acceptable practice, with only 20/176 participants scoring below the 80% total cutoff point (Table 2). Individual questions were answered correctly by 92.0% (162/176) to 99.4% (175/176) of participants, except for the first practice question, where only 71.6% (126/176) of participants reported participating in an infection control training program during the outbreak. Details of all the answers are summarized in Table 2.

Non-Emiratis and nurses, in general, had higher mean scores for practice compared to Emiratis (*U* = 1,461, *p* < 0.05) and physicians (*U* = 2,330, *p* < 0.05), as summarized in Table 4. Compared to nurses, physicians were less likely to participate in a training program (aOR: 0.37, 95% CI: 0.14–0.99, *p* < 0.05) and less likely to report a proper doffing of PPE (aOR: 0.19, 95% CI: 0.04–0.83, *p* < 0.05). Likewise, HCWs at fever clinics were less likely to report a proper doffing of PPE (aOR: 0.18, 95% CI: 0.04–0.65, *p* < 0.05).

## Discussion

Our study highlights several challenges facing HCWs working in primary healthcare centers in Dubai during the COVID-19 pandemic. First, the knowledge gap is quite vast, with only 57.4% (101/176) scoring a sufficient level of knowledge, even though the country had already passed the peak of the first outbreak wave by the time the study was conducted (7). In additional research in Vietnam, HCWs in a hospital setting exhibited a higher knowledge level (over 88% had adequate knowledge) even though it was conducted earlier in the pandemic (11). While direct comparison cannot be made confidently between the two studies, as Vietnam has a different healthcare setting and social background, and their research was conducted at a hospital level, we still speculate some of the differences in knowledge stem from two main reasons: one is the rapid change in recommendations and evidence related to the disease (18). Second is the infodemic phenomenon that has led to a campaign of misinformation and anecdotal evidence that is widespread amongst the public and HCWs (19–21). For instance, we recognize a pattern in the knowledge section that when the participants were asked about facts that either kept on changing as the evidence changed or had misinformation circulating related to it, they did not score high enough. Such examples include wearing masks by the general public, the isolation period for contact with a positive case, and antibiotic use as a treatment. At the beginning of the pandemic the recommendation was not to wear masks by the general public unless the person was symptomatic to help save PPE shortages. However, afterward, there was an emphasis locally and by the WHO about the importance of wearing face masks by the public to curtail the disease's spread by positive asymptomatic cases (22, 23). The quarantine of contacts and incubation period of the virus was not very clear to start with either. Subsequently the recommendation was 10–14 days in most countries, including the UAE (3, 24, 25). Finally, antibiotics, such as azithromycin, were not supported by any substantial evidence but were encouraged by various HCWs in different parts of the world (26, 27). Nevertheless, recent evidence refuted the benefits of such treatments (28).

We speculate that the evidence-based medicine (EBM) culture is weak amongst the HCWs population studied. Only 38.1% reported using scientific journals or research papers as their primary sources of information during the outbreak. Previously published data on EBM also highlighted such a weakness in the PHC sector in Dubai (29). This is alarming, as HCWs are considered frontline fighters against the disease and the misinformation campaign. The weaker their knowledge, the less likely they will be able to fight this misinformation. Furthermore, they could be the ones who are unintentionally spreading it. For example, when asked about the scientific name of the virus, which the WHO finalized, only 45% recognized that SARS-CoV-1 was incorrect and should have been SARS-CoV-2. Additionally, only 4 HCWs recognized that PCR does not analyze the virus's protein but rather its genetic material. This again is noteworthy, as two of the studied clinics were fever clinics that do COVID-19 PCR swabs daily.

In the attitude section, only 33% participants scored an adequate total score compared to similar studies (11, 16). Primarily, there was extreme concern about being infected or having a relative being infected by the virus. This type of concern was further addressed and highlighted in the literature by Shanafelt et al. as a multifactorial issue involving matters related to the organization's preparedness and its ability to protect its workforce's physical and mental well-being (30). Moreover, there were hesitations amongst the study subjects regarding taking the vaccine, being isolated in a healthcare facility if infected and agreeing that the pandemic would be successfully controlled. Once more, as highlighted earlier in the section, having such a low attitude makes it challenging to fight the widespread circulating misinformation about the disease, and such HCWs might unintentionally help in exacerbating the spread of such misinformation. Additionally, they might challenge maintaining a healthy workforce in the prolonged fight of the pandemic. Furthermore, with about 41% of the participants showing some degree of hesitation to take the vaccine, this highlights an important area for further exploration to overcome any future challenges in implementing a successful vaccination campaign, as highlighted by various studies in developing and developed countries (31–34). On the other hand, there was a greater positive willingness to continue fighting on the frontlines, with about 94% willing to do so in the future. This is a reassuring sign if further resurgence of the infection occurs in the future, as predicted by some experts (8, 9, 35).

While the HCWs in the study reported a high level of good infection control practice measures, they also highlighted the need to increase training programs regarding the disease during this pandemic, with only 72% (126/176) always participating in such programs during the outbreak. With further training and increased competency of HCWs, the staff's anxiety likely would be further alleviated, and the healthcare outcomes of the patients would be improved (30).

### Study Limitations

Our study exhibits an inherent recall bias that is expected in most cross-sectional questionnaire-based studies. Additionally, the attitude and practice sections could have been captured more accurately via interviewing the participants. The practice section does not necessarily reflect the actual practice of the HCWs, as they are reports by the participants. Thus, observing and auditing the participants' approach would provide further depth in this section. Our study noted that male participants constituted only 10% (18/176) of the study subjects, and Emirati participants were primarily physicians. This is not a pure selection bias issue but an actual reflection of the constituents of the workforce that makes the public primary healthcare sector in Dubai with a predominantly female workforce. Consequently, the results might not accurately reflect the views of the male HCWs or the Emirati nurses' KAP. Furthermore, the study only evaluated the views of family physicians and nurses in only four PHCs. As such, extrapolating the data to other HCWs and centers should to be taken into consideration. Moreover, the study was not powered to detect the differences between the various groups.

## Conclusion

As the world continues to battle the pandemic of COVID-19, evaluating the current KAP of the healthcare workforce regarding COVID-19 is paramount for winning this battle. The present study assessed the KAP of HCWs in the PHC sector in Dubai in fever and regular clinics. Significant gaps were identified regarding the virus's name, the type of testing, the transmission, and the isolation period of contacts with positive cases. Knowledge regarding signs and symptoms and the at-risk groups was generally satisfactory. Promotion of EBM culture amongst the HCWs in the PHC sector is greatly recommended to overcome the challenge of the infodemic phenomenon associated with COVID-19 and the related misinformation that is widely circulating worldwide. There was a significant level of anxiety amongst the study participants regarding contracting the disease themselves or their relatives and some degree of hesitancy to take the vaccine once it became available. We recommend that further training be provided to the HCWs to increase their confidence in battling the current outbreak and preparing them for any future surges of the disease. Vaccination hesitancy warrants further study and evaluation to overcome any additional obstacles by the time the vaccine becomes available. While most of the study participants reported performing satisfactory infection control measures, physical auditing needs to be evaluated at the workplace to ensure that corrective actions are in place and are being followed. Findings from our study warrant further similar exploration in the region and developing countries as similarities in the healthcare system setting and challenges might be shared. Not only might they apply to the current COVID-19 pandemic, but they will also help the region in overcoming and effectively handling any future pandemics and health emergencies.

## Data Availability Statement

Raw data for the results are accessible at: https://figshare.com/s/27f23a6fd98149070a67.

## Ethics Statement

The studies involving human participants were reviewed and approved by Dubai Scientific Research Ethics Committee at Dubai Health Authority. The patients/participants provided their written informed consent to participate in this study.

## Author Contributions

AHA was responsible for data analysis and writing the draft of the paper. All authors contributed to the review of the draft manuscript, subsequent modifications, design of the study, and data collection. All authors reviewed the manuscript before submission and approved the final version.

## Conflict of Interest

The authors declare that the research was conducted in the absence of any commercial or financial relationships that could be construed as a potential conflict of interest.

## Publisher's Note

All claims expressed in this article are solely those of the authors and do not necessarily represent those of their affiliated organizations, or those of the publisher, the editors and the reviewers. Any product that may be evaluated in this article, or claim that may be made by its manufacturer, is not guaranteed or endorsed by the publisher.

## Abbreviations

CI: confidence interval
COVID-19: coronavirus disease 2019
DHA: Dubai Health Authority
EBM: evidence-based medicine
HCW: healthcare worker
KAP, knowledge, attitude: and practice
PHC: primary healthcare center
PPE: personal protective equipment
SARS-CoV-2: severe acute respiratory syndrome-coronavirus-2
SD: standard deviation
UAE: United Arab Emirates
WHO: World Health Organization.
