# Supplementary material for: Knowledge, Attitude, and Practice Regarding COVID-19 Among Healthcare Workers in Primary Healthcare Centers in Dubai: A Cross-Sectional Survey, 2020
Source: Front Public Health. 2021 Jul 28;9:617679. doi: 10.3389/fpubh.2021.617679 (PMC8355417; doi:10.3389/fpubh.2021.617679)
Supplement: Supplementary file 1 [file Data_Sheet_1.PDF]

Knowledge, Attitude, and Practice Regarding COVID-19 among Healthcare Workers in Primary Healthcare Centers in Dubai, July 2020, a Cross-Sectional Survey

This study aims at evaluating your knowledge, attitude, and practice towards COVID-19 infection and outbreak. It comprises 4 sections: demographic information, knowledge, attitude, and practice regarding COVID-19. Try answering them to the best of your knowledge. Your participation in this survey is anonymous and all information will be kept confidential.

Demographics

For each of the following questions tick one answer that applies to you:

Gender:

- Male
- Female

Age group:

- <30
- 30-34
- 35-39
- 40-44
- ≥45

Nationality:

- Emirati
- Other

Profession:

- Nurse
- Physician

Years of experience as healthcare professional:

- <10
- 10-15
- >15

Current workplace:

- Fever clinic
- Regular primary health center (PHC)

Your primary source of information on COVID-19 is: (tick all that apply)

- Governmental and International health organizations websites and media, e.g. Ministry of Health, WHO and CDC
- Social media, e.g. WhatsApp, Facebook
- News media, e.g. TV, radio, newspaper
- Scientific journals and research papers
- Work colleagues
- Seminars and workshops
- Internet
- Other

Knowledge

Answer the following questions to the best of your knowledge.

Q1. There is currently no effective cure for COVID-19, but early symptomatic and supportive treatment can help most patients recover from the infection.
True False I don't know

Q2. Not all persons with COVID-19 will develop severe cases. Those who are elderly, have chronic illnesses, and are obese are more likely to be severe cases.
True False I don't know

Q3. Persons with COVID-19 cannot transmit the virus to others when a fever is not present.
True False I don't know

Q4. The COVID-19 virus spreads via respiratory droplets of infected individuals.
True False I don't know

Q5. Wearing general medical masks by the public can prevent one from acquiring infection by the COVID-19 virus.
True False I don't know

Q6. It is not necessary for children and young adults to take measures to prevent the infection by the COVID-19 virus.
True False I don't know

Q7. To prevent the infection by COVID-19, individuals should avoid going to crowded places such as bus parks and avoid taking public transportation.
True False I don't know

Q8. Isolation and treatment of people who are infected with the COVID-19 virus are effective ways to reduce the spread of the virus.
True False I don't know

Q9. People who have contact with someone infected with the COVID-19 virus should be immediately isolated in a proper place. In general, the observation period is 28 days.
True   False   I don't know

Q10. Diarrhea is a possible symptom of COVID-19.

True   False   I don't know

Q11. Currently COVID-19 vaccine is available in the market.

True   False   I don't know

Q12. Healthcare workers are at a higher risk of infection.

True   False   I don't know

Q13. Early antibiotic use shortens the duration of COVID-19 illness.

True   False   I don't know

Q14. SARS-CoV-1 is the causative agent of COVID-19 infection.

True   False   I don't know

Q15. Detection of the viral protein via PCR analysis of the patient's sample is the main way of diagnosing COVID-19.

True   False   I don't know

Attitude

For each of the following statements, mark if you agree, disagree, or undecided about your opinion on the statement.

A1. You are extremely worried that you might catch the COVID-19 infection.

Agree Disagree Undecided

A2. You are extremely worried that one of your family members might get infected.

Agree Disagree Undecided

A3. If getting COVID-19, you will accept isolation in health facilities.

Agree Disagree Undecided

A4. Prevalence of COVID-19 can be reduced by the active participation of healthcare workers in infection control programs.

Agree Disagree Undecided

A5. If a COVID-19 vaccine was available, I would have it.

Agree Disagree Undecided

A6. COVID-19 pandemic will be successfully controlled.

Agree Disagree Undecided

A7. If the country needs you, you will be willing to help in the frontline rescue.

Agree Disagree Undecided

Practice

For each of the following questions, state how frequently you participated in the activity.

P1. During the outbreak, did you participate in training programs to increase/refresh your practice on infection control and COVID-19?

Always Occasionally Never

P2. During the outbreak, did you use sodium hypochlorite or 70% alcohol as surface disinfectant?

Always Occasionally Never

P3. During the outbreak, did you wash your hands before and after contact with your patients?

Always Occasionally Never

P4. During the outbreak, did you maintain social distance at work place?

Always Occasionally Never

P5. During the outbreak, did you follow the steps in doffing your PPE as per protocol?

Always Occasionally Never

P6. During the outbreak, did you wear surgical mask for routine patient contact?

Always Occasionally Never
